# Editors’ Message March 2023

**DOI:** 10.1016/j.ajpc.2023.100490

**Published:** 2023-03-11

**Authors:** Nathan D. Wong, Erin D. Michos

**Affiliations:** a Division of Cardiology, University of California, Irvine School of Medicine, USA; b Division of Cardiology, Johns Hopkins University School of Medicine, USA

It is hard to believe 3 years have gone by and we are now beginning in 2023 our fourth year of publication of the American Journal of Preventive Cardiology! We wanted to begin by thanking the many authors, including key thought leaders in preventive cardiology and their junior colleagues and trainees who have contributed high quality original research, state-of-the-art reviews, and ASPC clinical practice statements over the past 3 years to help position our journal as among the top journals in the field. Without your trust and faith in the Journal, we would not be able to get to the place we are today. Importantly, the hard work and dedication of our associate editors and editorial board in ensuring the efficient and fair review of our submissions has been crucial to the success of the Journal and we owe much gratitude to them! Finally, we are grateful for the close collaboration and support from the staff and leadership of the American Society for Preventive Cardiology!

We are pleased to announce the expansion of our editorial board by first welcoming Karol Watson, MD, PhD and Michael Wilkinson, MD as new associate editors for the Journal. In addition, we welcome new editorial board members Monica Acevedo, MD, Mahmoud Al Rifai, MD, Elizabeth Dineen, DO, Andrew Freeman, MD, Wolfgang Koenig, MD, Puja Mehta, MD, Anum Saeed, MD, and Seamus Whelton, MD. We are hopeful these added experts to our editorial board will broaden the expertise and further streamline the review process.

In this issue, we want to highlight several key articles. First, we want to point out our first joint society statement “The importance of low-density lipoprotein cholesterol measurement and control as performance measures: A joint clinical perspective from the National Lipid Association and the American Society for Preventive Cardiology” written by authorship representing both the American Society for Preventive Cardiology and the National Lipid Association. We hope this represents the beginning of more collaboration between our like-minded societies as well as further joint publications with other societies as well. In addition, we are pleased to feature commentaries on advanced subclinical atherosclerosis as a novel risk category as well as on non-medical switching as a threat to patient care. Key state-of-the-art reviews for this issue include ones on imaging of subclinical atherosclerosis as a guide to lipid management, calcium scoring for risk stratification in South Asian persons, management of hypertension in sleep apnea, and a call to action for understanding differences between SGLT-2 inhibitors and GLP-1 receptor agonists. More than a dozen research articles are also featured in this issue, including but not limited to ones on acculturation and cardiovascular risk factors in Asian-American subgroups, cardiovascular health trajectories and mortality, AHA's Life's Essential 8 and cardiovascular health in young adults, relation of aldosterone and ideal cardiovascular health with incident diabetes, and older adult preferences regarding benefits and harms of statin and aspirin use in primary prevention.

We remain optimistic about the future of the Journal, but we want to hear from you, our readership, as well as our contributors about how we can further improve the Journal, what new topics and features you think could be added, how you think the Journal can best represent the scope and diversity of the field of Preventive Cardiology, and most importantly, serve you as the readers of the Journal. We again thank you for your patronage and support and look forward to a bright 2023!

Nathan D. Wong, PhD, MPH and Erin D. Michos, MD, MHS

